# Real electronic signal data from particle accelerator power systems for machine learning anomaly detection

**DOI:** 10.1016/j.dib.2022.108473

**Published:** 2022-07-16

**Authors:** Majdi I. Radaideh, Chris Pappas, Sarah Cousineau

**Affiliations:** Spallation Neutron Source, Oak Ridge National Laboratory, Oak Ridge, TN 37830, United States.

**Keywords:** Machine Learning, High voltage converter modulators, Digital signal processing, Anomaly detection, Spallation neutron source, Fault classification

## Abstract

This article describes real time series datasets collected from the high voltage converter modulators (HVCM) of the Spallation Neutron Source facility. HVCMs are used to power the linear accelerator klystrons, which in turn produce the high-power radio frequency to accelerate the negative hydrogen ions (H^−^). Waveform signals have been collected from the operation of more than 15 HVCM systems categorized into four major subsystems during the years 2020-2022. The data collection process occurred in the Spallation Neutron Source facility of Oak Ridge, Tennessee in the United States. For each of the four subsystems, there are two datasets. The first one contains the waveform signals, while the second contains the label of the waveform, whether it has a normal or faulty signal. A variety of waveforms are included in the datasets including insulated-gate bipolar transistor (IGBT) currents in three phases, magnetic flux in the three phases, modulator current and voltage, cap bank current and voltage, and time derivative change of the modulator voltage. The datasets provided are useful to test and develop machine learning and statistical algorithms for applications related to anomaly detection, system fault detection and classification, and signal processing.

## Specifications Table

**Table:** 

Subject	Electrical and Electronic Engineering
Specific subject area	Applied Machine Learning, signal processing, anomaly detection
Type of data	Table (time series)
How the data were acquired	The system controller collects waveform signals with a sampling rate of 400 ns and writes them to a hard drive on the controller's computer.
Data format	Analyzed
Description of data collection	The raw data from the controller were preprocessed to remove erroneoussignals that look like white noise. The relevant pulses with time lengthof 1.8 ms were extracted from the waveform to remove the timestamps when the system is idle, which significantly reduces data size.
Parameters of data collection	Time series data are collected from real-time operation of 15 different high voltage converter modulator systems during the period 2020-2022. Each system features 14 unique waveforms with either normal or anomaly signals.
Data source location	Spallation Neutron Source, Oak Ridge National Laboratory, Oak Ridge, Tennessee, United States.
Data accessibility	The data are presented in the Mendeley data repository with the following information: • Title of the repository: Real Electronic Signal Data from Particle Accelerator Power Systems for Machine Learning Anomaly Detection. • URL: https://data.mendeley.com/datasets/kbbrw99vh8/5 • DOI: https://doi.org/10.17632/kbbrw99vh8.5
Related Research Article	• M. I. Radaideh, C. Pappas, J. Walden, D. Lu, L. Vidyaratne, T. Britton, K. Rajput, M. Schram, S. Cousineau, Time series anomaly detection in power electronics signals with recurrent and convlstm autoencoders, preprint available at SSRN 4069225. http://dx.doi.org/10.2139/ssrn.4069225 • G. Pappas, D. Lu, M. Schram, D. Vrabie, Machine Learning for Improved Availability of the SNS Klystron High Voltage Converter Modulators, in: Proc. IPAC’21, no. 12 in International Particle Accelerator Conference, JACoW Publishing, Geneva, Switzerland, 2021, pp. 4303–4306. https://doi.org/10.18429/JACoW-IPAC2021-THPAB252

## Value of the Data

• These data provide quality signal data from the operation of the power systems in the spallation neutron source, which provides the most intense pulsed neutron beams in the world. These data are valuable to aid in the development of better algorithms for anomaly detection and fault type classification to reduce downtime in particle accelerators.
• Beneficiaries of these data include researchers, engineers, and instructors interested in machine learning, signal processing, and particle accelerator physics.
• These data provide a baseline for assessment and designing optimal machine learning algorithms for anomaly detection, fault classification, signal processing, and time series modeling.

## 1 Data Description

**Fig. 1:**
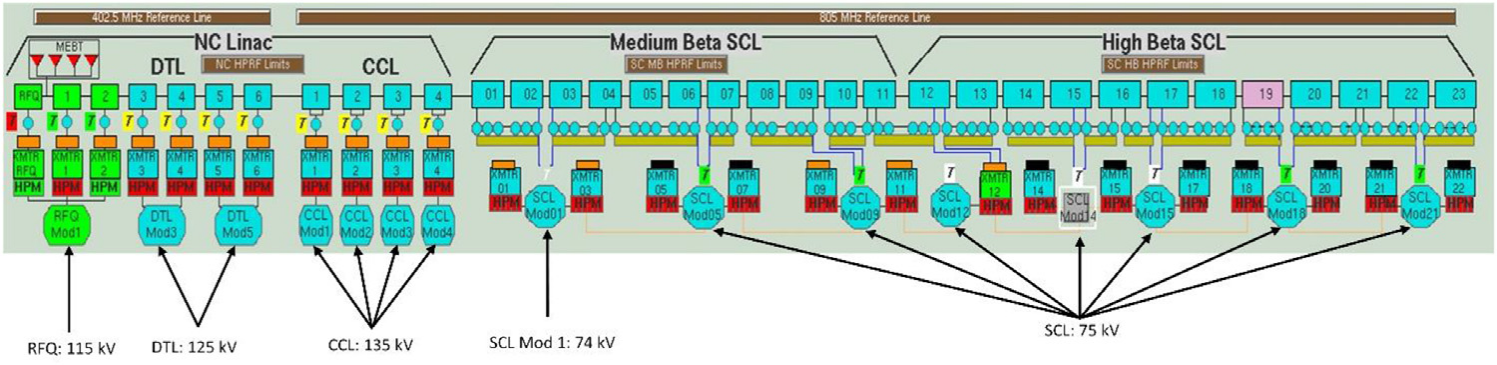
Layout of the HVCM systems in the SNS.

High Voltage Converter Modulators (HVCM) continue to have frequent failures, making them a major source of down time for the Spallation neutron source. HVCMs are well instrumented to collect large amounts of waveform data including but not limited to modulator current, modulator voltage, magnetic flux, cap bank voltage, cap bank current, and others. These waveform data are collected from all 15 HVCM systems (see Fig. 1) for the application of anomaly detection and failure prediction. All 15 modulators are grouped into four major subsystems based on their type as follows: 1 modulator as radio-frequency quadrupole (RFQ), 2 modulators as drift-tube linac (DTL), 4 modulators as coupled-cavity linac (CCL), and 8 modulators as super-conducting linac (SCL). A waveform is featured by consecutive pulses, which could be either a normal pulse if the system is healthy, or anomaly pulse if the system is close to failing. These pulses are collected and reported in the dataset of this paper, where the details of the data collection and processing are described in the next section.

The dataset repository has a total of 10 files: 8 binary data files, 1 Python script, and 1 Excel sheet containing excerpts of readable data from the binary files. All files are listed and described in Table 1. Each system (RFQ, CCL, DTL, SCL) has two data files, one containing the waveform data, and one containing the labels of these waveforms. Each waveform data file comprises a 3D numpy array, with the axes explained as follows

$$\mathrm{shap}e^{x}=\left(N_{\mathrm{puls}\mathrm{es}}\times N_{\mathrm{times}}\times N_{\mathrm{feat}\mathrm{ures}}\right)$$

where $N_{pulses}\;$is the number of different pulses/samples collected from the system, $N_{times}=4500\;$is the number of time steps for each pulse, and $N_{features}=14$ is the number of different features or waveform types recorded for each pulse (e.g. magnetic flux, modulator voltage, modulator current). The waveform file contains both normal and faulty waveforms, where the label can be identified via the labels' dataset, which is a 2D numpy array with the axes explained as follows:

$$\;\;\;\;shape^{y}=\;\left(N_{pulses}\times N_{labels}\right),\;$$

where $N_{pulses}$ must match the value in $shape^{x}$ and $N_{labels}=3$ are the three labels/columns which are respectively: **index, status**, and **type**. The “**index**” is a string path value that indicates the exact origin of the pulse, for example, whether it belongs to DTL3, DTL5, CCL1, SCL1, etc. as illustrated for the 15 modules in Fig. 1. This gives the user an easy way to re-categorize the data in case the user is interested in a single modulator analysis. The second label, “**status**”, is basically used for binary classification, and can be either “Run” or “Fault”. The third label, “**type**”, is used for multi-class classification, as it shows more detailed info about the status. If it is a “Run” pulse, the third column will show “Normal”, but if it is a “Fault” pulse, the fault source is given, e.g., fiber fault, - CB V Low Fault, SNS PPS Missing, DV/DT High Fault, B-* Driver Fault, and several others.

**Table 1:** Description of the data files shared in the repository.

File	Description	Shape
**RFQ.npy**	3D numpy array of the normal and faulty pulses for the RFQ system	(872, 4500, 14)
**RFQ_labels.npy**	2D numpy array of the labels for the RFQ system	(872, 3)
**DTL.npy**	3D numpy array of the normal and faulty pulses for the DTL system	(1077, 4500, 14)
**DTL_labels.npy**	2D numpy array of the labels for the DTL system	(1077, 3)
**CCL.npy**	3D numpy array of the normal and faulty pulses for the CCL system	(2057, 4500, 14)
**CCL_labels.npy**	2D numpy array of the labels for the CCL system	(2057, 3)
**SCL.npy**	3D numpy array of the normal and faulty pulses for the SCL system	(4598, 4500, 14)
**SCL_labels.npy**	2D numpy array of the labels for the SCL system	(4598, 3)
**load_dataset.py**	A simple python script to load and plot the dataset	NA
**sample_data.xlsx**	An excel file containing selected readable data to view based on **RFQ.npy** and **RFQ_labels.npy**	NA

The Python script (**load_dataset.py**) will show the user a simple way to load and interpret the dataset files before using them for different applications. The 14 waveforms are described in the next section.

Due to the large size of the dataset and the 3D shape of the waveform arrays, it is more convenient to save the data in a binary format to save disk space and reduce the number of data files. This also allows loading the data into its right shape for machine learning applications using **load_dataset.py**, which reduces the errors that may occur due to data manipulation of many files. However, for the reader's interest, we report some excerpts of the data in the readable excel file **sample_data.xlsx**, which gives the reader an impression of the binary data. This file contains three sheets described as follows:

1. **RFQ.npy_A+IGBT-I**: This sheet reports a 2D slice of the data in the **RFQ.npy** file, which corresponds to the IGBT current in the A phase (A+IGBT-I) for the RFQ. The shape of the data in this sheet is (872, 4500), where the rows represent the pulse number, and the columns represent the time steps.
2. **RFQ.npy_A-flux**: This sheet reports another 2D slice of the data in the **RFQ.npy** file, which corresponds to the magnetic flux in the A phase (A-Flux) for the RFQ. Similarly, the shape of the data in this sheet is (872, 4500), where the rows represent the pulse number, and the columns represent the time steps.
3. **RFQ_labels.npy:** This sheet reports the corresponding labels saved in the binary file **RFQ_labels.npy**. The shape of the data in this sheet is (872, 3), where the rows represent the pulse number, and the columns represent the **index, status, and type**.

The remaining waveform slices, label files, and other systems (DTL, CCL, SCL) follow the same structure.

## 2 Experimental Design, Materials and Methods

The Spallation Neutron Source (SNS) at Oak Ridge National Laboratory provides the most intense pulsed neutron beams in the world [1]. During SNS operation, negative hydrogen ions are accelerated to very high speeds (about 90% of the speed of light) before hitting a carbon foil to produce high energetic protons. The proton beam then strikes a mercury target to cause a spallation reaction; generating neutron beams for scientific research. As part of this process, HVCM systems are used to power the linear accelerator klystrons. There is a total of 15 HVCM systems at the SNS driving a total of 92 klystrons [2]. Fig. 1 shows the layout of the 15 HVCM systems at the SNS. The 15 modulators are grouped into four major subsystems based on their operating voltage as follows: RFQ (1 modulator) at 115 kV, DTL (2 modulators) at 125 kV, CCL (4 modulators) at 135 kV, and SCL (8 modulators) at 74-75 kV.

**Fig. 2:**
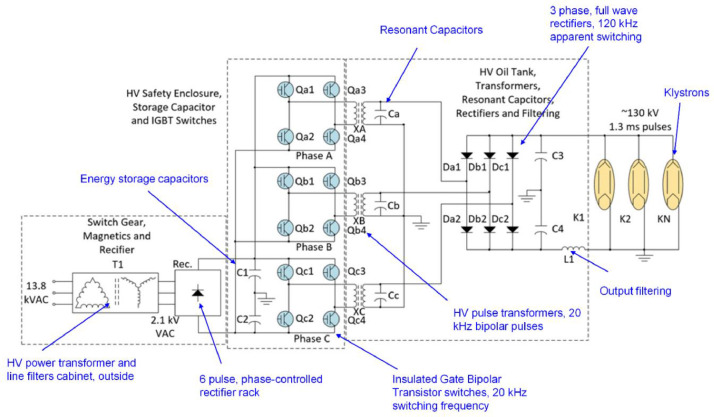
A simplified schematic of the HVCM circuit.

HVCM systems are used to convert 3ϕ 13.8 kVAC into a train of up to 135 kV, 1.3 ms pulses at 60 Hz to klystrons, which power the accelerating cavities at the SNS. A simplified schematic of the HVCM parts is shown in Fig. 2, which can be summarized into these subsystems:

• AC switch gear and magnetics.
• A six-pulse phase-controlled rectifier unit, which converts the 2100 VAC to ±0-1300 VDC.
• The insulated-gate bipolar transistor (IGBT) switches labeled Qa1 to Qc4 in Fig. 2 are used to chop the DC voltage at the cap banks at 20 kHz, stepping the chopped voltage up to high voltage, and then rectifying and filtering the high voltage to klystrons.
• HVCM controller rack for triggering, machine protection, and interlocks.

The HVCMs at the SNS have the same circuit topology and are operated in a similar fashion. However, the differences originate in the frequency modulation, operating conditions, and design values such as klystron perveance, operating voltage, turns ratio, leakage Inductance, and capacitor values. For example, DTL operates at 125kV while SCL operates at 75kV. Also, RFQ has leakage Inductance of 9µH while SCL has 5µH.

### 2.1 Data collection

The HVCM controller monitors up to 32 waveforms from the modulator, we only report the most useful 14 waveforms that experts believe they can be useful for machine learning and anomaly detection applications. The normal waveform signals are digitized at 50 mega-sample per second (MS/s). For faulty waveforms, they are saved permanently at 50 MS/s with a record length of 3 ms centered on the pulse where the fault occurs, and another file at 2.5 MS/s and a record length of 36 ms. A settings file is saved whenever a change is made in the HVCM settings, but these setting files are not reported in this dataset as tuning does not occur frequently. All data are collected using LabVIEW and saved in a CSV (comma-separated values) format.

The 14 waveforms (features) reported in the dataset **in order** are:

1. A+IGBT-I: The current passing through the IGBT switch of phase A+ in **Qa1** in Fig. 2 (unit: A).
2. A+*IGBT-I: The current passing through the IGBT switch of phase A+* in **Qa3** in Fig. 2 (unit: A).
3. B+IGBT-I: The current passing through the IGBT switch of phase B+ in **Qb1** in Fig. 2 (unit: A).
4. B+*IGBT-I: The current passing through the IGBT switch of phase B+* in **Qb3** in Fig. 2 (unit: A).
5. C+IGBT-I: The current passing through the IGBT switch of phase C+ in **Qc1** in Fig. 2 (unit: A).
6. C+*IGBT-I: The current passing through the IGBT switch of phase C+* in **Qc3** in Fig. 2 (unit: A).
7. A-Flux: Magnetic flux density for phase A in transformer **XA** in Fig. 2 (unit: -).
8. B-Flux: Magnetic flux density for phase B in transformer **XB** in Fig. 2 (unit: -).
9. C-Flux: Magnetic flux density for phase C in transformer **XC** in Fig. 2 (unit: -).
10. Mod-V: Modulator voltage (unit: V).
11. Mod-I: Modulator current (unit: A).
12. CB-I: Cap bank current (unit: -).
13. CB-V: Cap bank voltage (unit: V).
14. DV/DT: Time derivative change of the Mod-V voltage (unit: -).

To allow easier view of the waveforms on the screen for the operators, certain waveforms were scaled by the controller, where their absolute unit is not provided above. For example, the magnetic flux (A-Flux, B-Flux, C-Flux) reported in the dataset is multiplied by a factor of about 15. Therefore, no unit is given for these waveforms to preserve the raw data.

The magnetic flux in the transformer cores is measured with a Rogowski coil, model RCTi3ph with an accuracy of ±1%. The output from the Rogowski coil is integrated with an operational amplifier integrator before digitizing the waveform. IGBT currents are measured with a LANL-designed (Los Alamos National Laboratory) Rogowski coil around the primary feeds from each leg of the H-bridges. Modulator high voltage (Mod-V) is measured with a North Star High Voltage model VD-120B pulse compensated divider, and the modulator current (Mod-I) is measured with a Stangenes current transformer model SI-014463.

### 2.2 Data processing

We tried to limit data processing steps in this work so that users have a very close form to raw data, giving them more flexibility to apply their own smoothing and scaling techniques. All processing and scripting were done in Python. First, we remove erroneous samples from the raw data, which look like a white noise. These samples are not useful as they do not carry information, therefore, they have been removed.

The next step is crucial for data size reduction given that the sampling rate is high (400 ns). We extract 1.8 ms pulses from a waveform of 36 ms length, that typically has 3 pulses. The idle time between the pulses is cut from the waveform to significantly reduce data size, given that the idle time is not useful for anomaly detection, and the system will be off during this time preparing for the next pulse. Each 1.8 ms pulse has 4500 time steps (i.e. sampling rate is 400 ns). It is worth highlighting that extracting the pulses from the raw waveform can reduce the total data size from 18 GB to 2.1 GB.

Next, we group the pulses from similar HVCM systems into a representative category to reduce the number of files and categories in the dataset. For example, all pulses from CCL1, CCL2, CCL3, and CCL4 modulators are grouped into CCL, and so on for DTL and SCL. The final data arrays are saved into a numpy binary file as indicated in Table 1. Afterward, the users can apply smoothing, filtering, and scaling techniques to these pulses as appropriate for the application of interest.

**Fig. 3:**
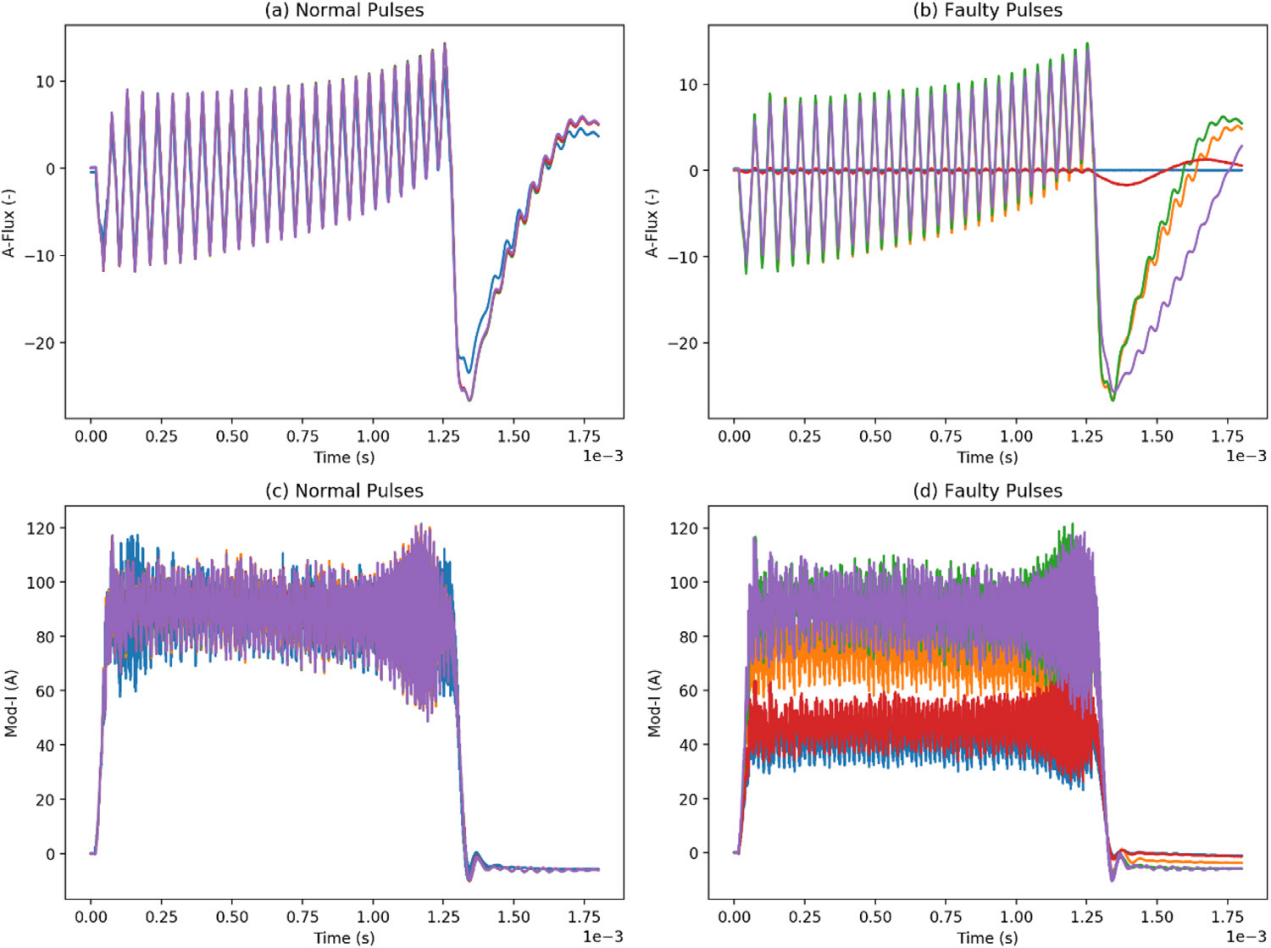
Five selected pulses from the RFQ: (a) normal magnetic flux in the A phase, (b) faulty magnetic flux in the A phase, (c) normal modulator current, (d) faulty modulator current.

Fig. 3 shows a plot of 5 randomly selected normal and faulty pulses in the RFQ system for two different waveforms: A-flux (magnetic flux in the A phase transformer) and Mod-I (modulator current). As can be seen in Fig. 3(a) and (c), the normal pulses tend to exhibit a similar trend. However, by looking at Fig. 3(b) and (d), it can be noticed that faulty waveforms can still look like normal as is the case for faulty A-flux pulses in Fig. 3(b), or obviously anomalous as is the case for Mod-I in Fig. 3(d). Either way this shows the value of relying on multivariate anomaly detection through analyzing multiple waveforms to detect the system anomaly depending on the cause, as the fault can be obvious in some waveforms more than the others.

### 2.3 Previous data usage

Parts of this dataset were used in different machine learning studies in the past. For example, anomaly detection was applied to some of the SCL waveform data using discrete cosine transform, showing good results given the limited data available at that time [3]. Recently, an application of recurrent neural network autoencoders was demonstrated on signals from the RFQ module [4]. The authors developed autoencoder models based on bi-directional gated recurrent unit, bi-directional long-short term memory (LSTM), and convolutional LSTM (ConvLSTM), which all demonstrated very good precision and recall metrics compared to other classical machine learning methods.

## Ethics Statements

All methods were carried out in accordance with guidelines and regulations at the Oak Ridge National Laboratory. In addition, the data were approved by the export control office at the Oak Ridge National Laboratory.

Notice: This manuscript has been authored by UT-Battelle, LLC, under contract DE-AC05-00OR22725 with the US Department of Energy (DOE). The US government retains and the publisher, by accepting the article for publication, acknowledges that the US government retains a nonexclusive, paid-up, irrevocable, worldwide license to publish or reproduce the published form of this manuscript, or allow others to do so, for US government purposes. DOE will provide public access to these results of federally sponsored research in accordance with the DOE Public Access Plan (http://energy.gov/downloads/doe-public-access-plan).

## CRediT Author Statement

**Majdi I. Radaideh:** Conceptualization, Methodology, Software, Data curation, Visualization, Formal analysis, Writing - Original Draft; **Chris Pappas:** Conceptualization, Software, Data curation, Writing – review & editing; **Sarah Cousineau:** Conceptualization, Funding acquisition, Resources, Writing – review & edit.

## Declaration of Competing Interest

The authors declare that they have no known competing financial interests or personal relationships that could have appeared to influence the work reported in this paper.

## Data Availability

Real Electronic Signal Data from Particle Accelerator Power Systems for Machine Learning Anomaly Detection (Original data) (Mendeley Data).
